# Roles and therapeutic opportunities of ω-3 long-chain polyunsaturated fatty acids in lung cancer

**DOI:** 10.1016/j.isci.2024.111601

**Published:** 2024-12-14

**Authors:** Jiang Luo, Shu Peng, Ziyu Jiang, Qingwei Wang, Mini Zhang, Yuxin Zeng, Yu Yuan, Min Xia, Zixi Hong, Yufei Yan, Yushuang Tan, Jiawen Tang, Conghua Xie, Yan Gong

**Affiliations:** 1 Department of Pulmonary Oncology, Zhongnan Hospital of Wuhan University, Wuhan, China; 2 Tumor Precision Diagnosis and Treatment Technology and Translational Medicine, Hubei Engineering Research Center, Zhongnan Hospital of Wuhan University, Wuhan, China; 3 Department of Thoracic Surgery, Tongji Hospital, Tongji Medical College, Huazhong University of Science and Technology, Wuhan, China; 4 Hubei Key Laboratory of Tumor Biological Behavior, Zhongnan Hospital of Wuhan University, Wuhan, China

**Keywords:** Health sciences, Natural sciences, Applied sciences

## Abstract

Over the past decades, researchers have continuously investigated the potential functions of long-chain polyunsaturated fatty acids (LCPUFAs) in cancers, including lung cancer. The ω-3 LCPUFAs, primarily consisting of eicosapentaenoic acid and docosahexaenoic acid, were found to modify inflammatory tumor microenvironment, induce cancer cell apoptosis and autophagy, and suppress tumor development when administered alone or with other therapeutical strategies. Although the precise anti-tumor mechanism has not been elucidated yet, ω-3 LCPUFAs are often used in the nutritional treatment of patients with cancer due to their ability to significantly improve patient’s nutritional status, increase the sensitivity of tumor cells to treatments, and alleviate cancer-related complications. Here we present the key roles of ω-3 LCPUFAs as dietary supplementations in lung cancer, comprehensively review the recent progress on the underlying mechanisms of cancer cell regulation by ω-3 LCPUFAs, and introduce the application of ω-3 LCPUFAs in the clinical management of lung cancer and its malignant complications.

## Graphical abstract

**Figure:**
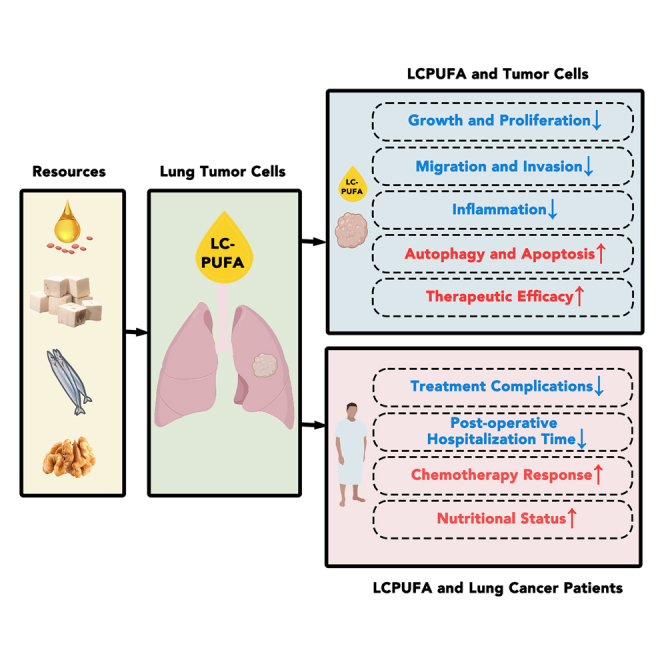

## Introduction

With an estimated 2 million new cases and approximately 1.8 million patient deaths each year, lung cancer is among the most lethal cancers.^1^ While immunotherapy and targeted therapies have dramatically improved lung cancer treatment, the five 5-year survival rates are still unsatisfied. Moreover, these strategies, as well as radiotherapy, chemotherapy, and surgical treatments have a number of negative symptoms,^2^^,^^3^ such as nausea, anemia, lack of appetite, and losing weight.^4^^,^^5^ Globally 45–69% of patients with lung cancer are reported to develop nutritional deficiencies, which might result in poor responses to therapeutical treatments and poor quality of life for patients.^6^^,^^7^ The European Society for Clinical Nutrition and Metabolism (ESPEN) encourages the early start of nutritional management for patients with cancer, considering that it is difficult to reverse weight and muscle wasting after the patient has become severely malnourished.^8^^,^^9^ Previous reports have also shown that lifestyles such as physical activity and diet, in addition to smoking, can influence a patient’s risk of lung cancer and that fatty acids (FAs) are the main dietary components that regulate the progression of lung cancer.^10^

FAs function as not only energy substances, but also major components of cell membranes,^11^^,^^12^ and act as signaling molecules involved in inflammation,^13^ immunity,^14^ and cell proliferation^15^ in the lungs. Numerous studies demonstrated that patients with lung cancer had significant abnormalities of lipid metabolism, including the biosynthesis of unsaturated FAs.^16^^,^^17^^,^^18^ Among them, is the consumption of different long-chain polyunsaturated FAs (LCPUFAs) in several prospective and case-control studies. Dan Lv et al. and Hung. N. Luu et al. discovered that a high level of LCPUFA intake reduced the risk of lung cancer,^19^^,^^20^ and Yu-Fei Zhang et al. revealed that a large amount of LCPUFA intake exacerbated the risk, while a small amount had no effect.^21^ However, Hang Zhao et al. identified a positive correlation between high levels of docosahexaenoic acid (DHA) and the risk of lung cancer.^22^ The discrepancies of these studies may result from differences in the types and amounts of LCPUFAs in the diet and dietary recall deficits.

**Figure 1:**
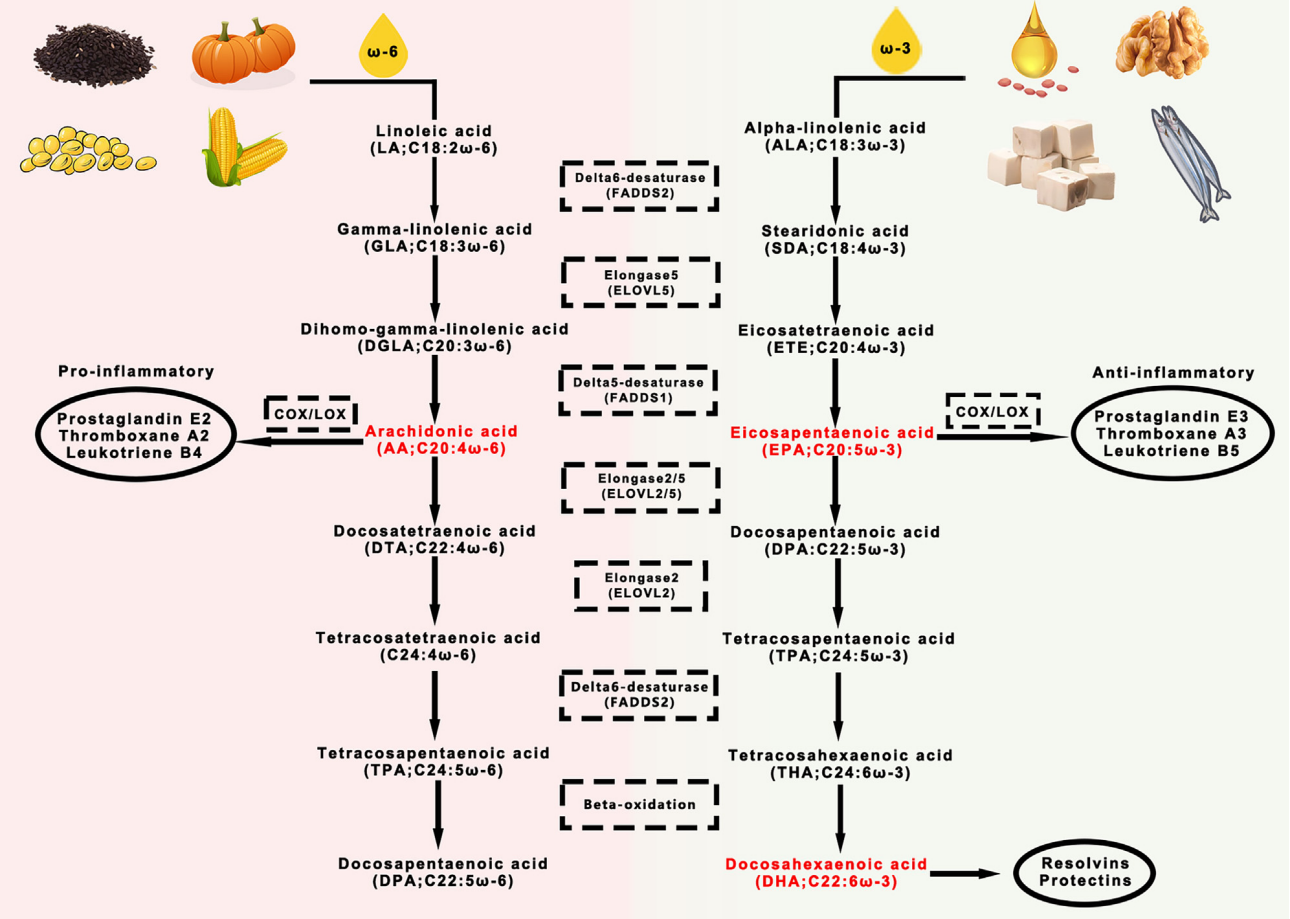
The major sources and synthesis of ω-6 and ω-3 LCPUFAs in the human body

Due to the lack of necessary fatty acid desaturase to convert oleic acid into linolenic acid, it is impossible for humans to endogenously synthesize LCPUFAs in adequate amounts.^23^ Therefore, they can only be obtained by exogenous routes such as foods, and are considered as essential FAs.^24^ With exogenous dietary supplementation, LCPUFAs can be converted into each other. For example, α-linolenic acid can be desaturated by the delta 5 and 6 desaturase enzymes and elongated by Elovl 2 and 5 elongases, transforming into DHA and eicosapentaenoic acid (EPA) in multiple organs (liver, brain, testicles, and so forth).^25^^,^^26^^,^^27^^,^^28^^,^^29^ These rate-limiting enzymes are influenced by LCPUFAs and redox status so as to keep LCPUFAs at appropriate levels (Figure 1).^27^ People with diets high in ω-3 LCPUFAs (such as Greenland) have lower cancer incidence than those with the Westerner diets often containing high amounts of ω-6 LCPUFAs.^30^^,^^31^ Increasing researches also showed that supplementation with ω-3 LCPUFAs benefited the therapeutical prognosis of patients with lung cancer.^6^^,^^32^^,^^33^

**Figure 2:**
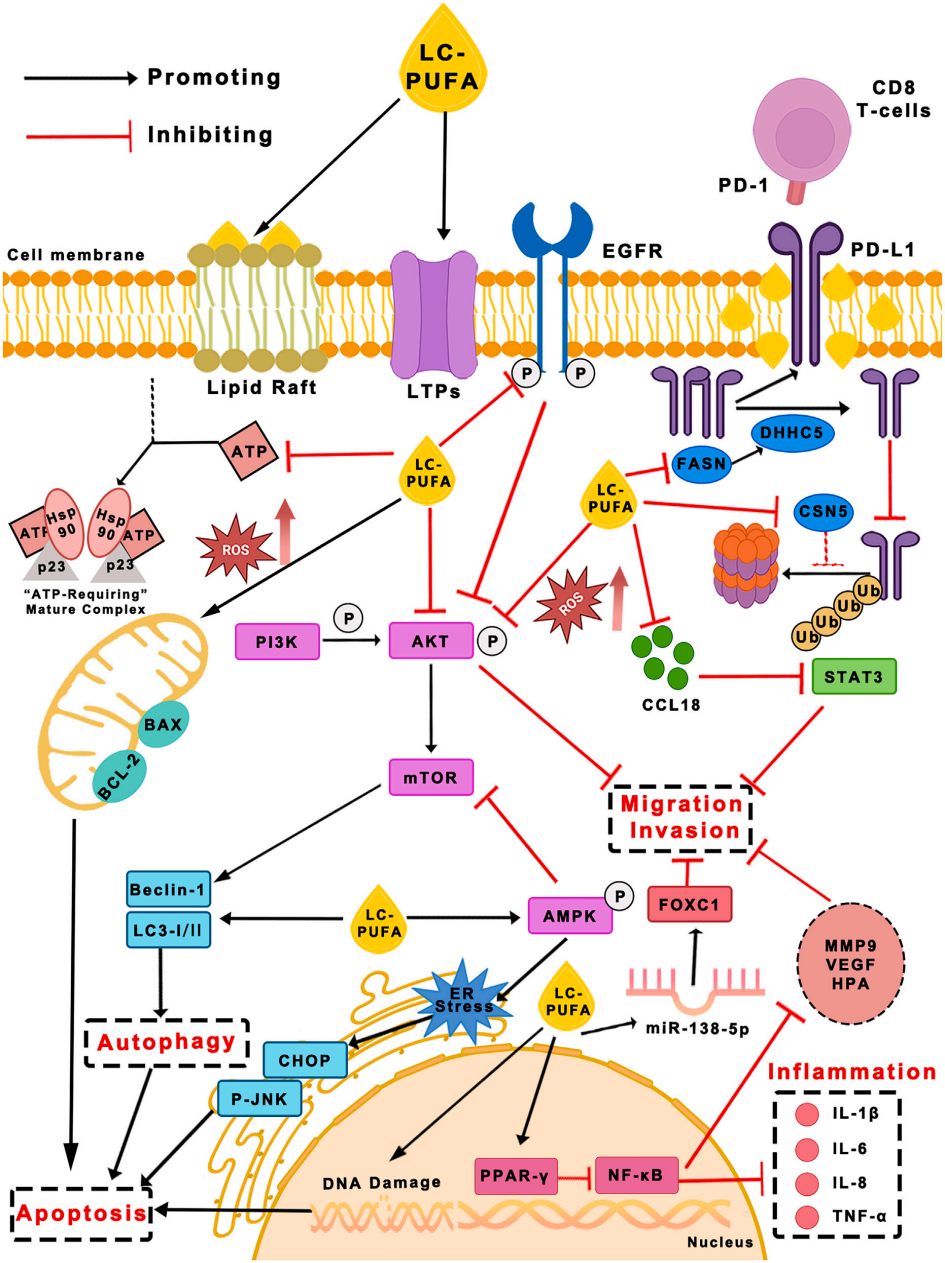
LCPUFAs regulate lung cancer progression via modulating lipid rafts on the membrane and multiple pathways involved in cell apoptosis, autophagy, migration, invasion, inflammatory factor production, and immunotherapy responses

This review aimed to provide an update on the progress of ω-3 LCPUFA studies in the pathogenesis and treatments of lung cancer. In general, we discuss the association between ω-3 LCPUFA supplementation and lung cancer progression, prevention, treatment, and prognosis, focusing on the evidence of ω-3 LCPUFAs’ effects and functional mechanisms (Figure 2) in lung cancer cells and animal models, as well as clinical outcomes of patients with lung cancer with different diets and nutritional supplementations.

## ω-3 long-chain polyunsaturated fatty acids decelerate lung cancer progression

Increasing researches reveal that ω-3 LCPUFAs present anti-tumor effects through various pathways, including the inhibition of cancer cell proliferation,^34^^,^^35^ tumor metastasis, angiogenesis, and inflammation, as well as the induction of cell-cycle arrest and apoptosis.^36^^,^^37^^,^^38^ Both *in vivo* and *in vitro* studies suggest that ω-3 LCPUFAs and their derivatives effectively prevent lung cancer progression via suppressing cancer cell viability and migration, as well as altering tumor inflammatory and immune microenvironment.^39^^,^^40^^,^^41^ The potential mechanisms are: 1) ω-3 LCPUFAs and their metabolites participate in and regulate lipid peroxidation and induce mitochondrial damage in neoplastic cells^42^^,^^43^^,^^44^; 2) ω-3 LCPUFAs and their metabolites, as ligands, bind to nuclear receptors such as peroxisome proliferator-activated receptors (PPARs) to regulate pro- or anti-oncogene expression and induce chromosome damages^31^; 3) ω-3 LCPUFAs and their metabolites regulate protein kinase C (PKC), nuclear factor-κB (NF-κB) and mitogen-activated protein kinase (MAPK) signaling pathways. We summarize the significant findings of ω-3 LCPUFAs in lung cancer since 2011 (Table 1).

**Table 1:** ω-3 LCPUFA effects and mechanisms in lung cancer

Effects	Cell lines or animal models	Compounds	Mechanism of action	Reference
Inhibit tumor/cancer cell growth and proliferation	LLC mouse model	DHA/EPA	Downregulate NF-κB pathway through PPARγ	Liu et al.^37^
	LLC cells	ω-3/ω-6LCPUFA diet	Enhance CYP450 metabolism	Huerta-Yepez et al.^45^
	A427 cells	DHA	Reduce TNFα expression via upregulating lipid peroxidation	Muzio et al.^46^
Induce autophagy and apoptosis	A549 cells	EPA/DHA	Upregulate lipid peroxidation and induce autophagy	Zajdel et al.^47^
	LLC mouse model	DHA	Regulate AKT/mTOR or AMPK pathway	Kim et al.^48^
	A549 cells	DHA	Regulate PI3K/AKT pathway	Yin et al.^49^
	95D cells	DHA	Upregulate PPARγ and RXRα signaling	Yue et al.^50^
	A549 cells	DHA/EPA	Activate the AKT/mTOR pathway and regulate autophagy	Yao et al.^51^
	A549 cells	DHA/EPA	Induce autophagosome formation	Yao et al.^52^
Reduce cancer cells migration and invasion	A549 cells	DHA	Inhibit HSP90 expression	Mouradian et al.^53^
	A549/95D cells	DHA	Inhibit CCL18/STAT3 signaling and EMT	Luo et al.^54^
	95D cells	DHA	Activate PPARγ and regulate NF-κB and PTEN/AKT pathways	Yin et al.^55^
	LLC cells	DHA	Enhance miR-138-5p expression and decrease FOXC1 expression	Bai et al.^56^
Suppress inflammation	A549 cells	PSO/RA-RF	Downregulate lipid peroxidation	Tantipaiboonwong et al.^57^
	A549/H1299 cells	EPA	Regulate COX metabolism	Yang et al.^58^
Increased tumor therapeutic efficacy	A549/H1573 cells	DHA	inhibit Ras/MEK/ERK and PI3K/AKT pathways to enhance diclofenac efficacy	Poku et al.^59^
	A549\H1299 cells	DHA	inhibit EGFR and ERK pathways to increase carboplatin’s effects	Morin et al.^60^
	PC9 cells	DHA	Regulate EGFR pathway to reverse gefitinib sensitivity	Ding et al.^61^
	LLC cells	DHA	Synergize with DTX to inhibit lung cancer metastasis to bone	Jiang et al.^62^
	A549/H1385/H1650/H1975 cells	DHA	Reduce membrane fluidity and increase PD-L1/PD-1 binding to increase the effects of ICIs	La Vecchia et al.^63^
	A549 cells	DHA	Reverse PD-L1-mediated immune suppression via decreasing the PD-L1 expression	Zhang et al.^64^

PSO/RA-RF: perilla seed oil/rosmarinic acid-rich fraction.

### ω-3 long-chain polyunsaturated fatty acids suppress lung cancer via inhibiting cell growth and cell proliferation

The fundamental biological capabilities of malignant tumors are maintenance of cell proliferation, resistance to cell death, activation of invasion, and metastasis. ω-3 LCPUFAs are capable of influencing the cell growth and proliferation ability of lung carcinoma cells.

Previous studies indicated that both ω-3 LCPUFA decreased lung cancer cell proliferation.^46^ Further researches revealed that the anti-proliferative effects of ω-3 LCPUFAs were achieved through several signaling pathways.^58^ The role of EPA and DHA on NSCLC cell proliferation were studied in cyclooxygenase (COX)-2 overexpressed A549 and COX-2 deficient H1299 cells by Yang P et al. Both EPA and DHA effectively suppressed A549 cell proliferation rather than H1299 cells, indicating that the COX-2 levels were responsible for the anti-proliferative functions of ω-3 LCPUFAs. Further studies revealed that prostaglandin (PG) E2 boosted NSCLC cell proliferation via upregulating the AKT/protein kinase B pathway, while PGE3 suppressed this pathway.^58^ In addition, they proved that the anti-proliferative mechanism of ω-3 LCPUFA was related to lipid peroxidation and PPAR activity.^6^^,^^65^ While PPARγ was reported to promote cycle arrest of cancer cells,^37^^,^^66^ Muzio G. et al. found that ω-3 LCPUFAs reduced lung cancer cell proliferation via inducing PPARγ.^46^

### ω-3 long-chain polyunsaturated fatty acids suppress lung cancer via promoting apoptosis

Besides inhibiting cell growth, inducing apoptosis in tumor cells has been a key strategy for tumor treatment, the cytotoxic effects of ω-3 LCPUFAs on cancer cells have been confirmed both *in vitro* and *in vivo*. A remarkable increase of apoptotic cells along with the percentage of G1-phase cells was found in A549 cells treated with DHA, indicating that DHA induces NSCLC cell apoptosis.^48^ Yuanyuan Liu et al. revealed that the levels of pro-apoptotic factors BAX and Caspase-3 were upregulated after DHA and EPA treatments while downregulating the anti-apoptotic factors Bcl-2 and Bcl-xl.^37^ Similarly, Alicja Zajdel et al. reported that both EPA and DHA decreased cellular viability, accelerated apoptosis via activating Caspase-3/7 in a concentration-dependent manner in A549 cells. Probably due to the higher unsaturation of DHA, the effects of DHA were more effective than EPA.^47^ These findings collectively indicated ω-3 LCPUFAs induced apoptosis via regulating the enzymatic activity of caspases, thereby preventing tumor development.

In addition, increasing evidence suggests that oxidative stress is extremely closely related to apoptosis.^47^^,^^67^ Recently, Payungsak Tantipaiboonwong et al. reported that perilla seed oil (PSO)/rosmarinic acid-rich fraction (RA-RF) antioxidants exerted anti-tumor effects via scavenging tumor necrosis factor (TNF)-α-induced reactive oxygen species (ROS) levels.^57^ It was believed that ω-3 LCPUFAs were susceptible to peroxidation and increased the levels of ROS produced by anti-tumor drugs.^43^ Many researches showed that the pro-apoptotic effects of DHA were dependent on the DHA-induced accumulation of ROS in tumors.^47^^,^^68^^,^^69^ In A459 cells, superoxide dismutase 1 and 2 levels were unchanged after exposure to DHA, but catalase expression was downregulated. DHA triggered apoptosis in neoplastic cells via increasing ROS production through the downregulation of catalase, while the antioxidant NAC reversed DHA’s effects.^49^ Meanwhile, Alicja Zajdel et al. discovered that EPA and DHA increased the intracellular concentration of oxidized DNA and proteins in A549 cells, while the antioxidant NAC abolished the inhibitory effects of ω-3 LCPUFAs cytotoxicity and Caspase-3/7 activation, indicating that the increased intracellular oxidative damage was responsible for the cell death caused by EPA and DHA.^47^

Moreover, Emeline Dierge et al. found that ω-3 LCPUFAs significantly induced iron concentration in cancer cells under ambient acidotic conditions, promoting cell death, which in turn significantly delayed tumor growth in mice.^70^ Pleasingly Kikawa et al. showed that DHA supplementation enhanced oxidative stress and carcinoma death, strengthening radiosensitivity of lung cancer.^71^ Furthermore, oxidative stress induced by ω-3 LCPUFAs could also regulate the signaling pathways related to ROS, such as MAPK, NF-κB or activator protein 1 transcription factors.^43^^,^^72^ Although the relationship between ROS and cell apoptosis needs to be further explored, the anti-tumor properties of ω-3 LCPUFAs that were mediated by ROS are confirmed.

### ω-3 long-chain polyunsaturated fatty acids suppress lung cancer via promoting autophagy

Autophagy has double-edged effects that can either promote or inhibit cell mortality. Inhibition of autophagy can increase sensitivity to apoptotic stimuli,^47^^,^^73^ and enhances mutations and causes oncogenesis.^49^ EPA and DHA-induced autophagy enhanced lung cancer cell apoptosis,^47^ and further investigations indicated that ω-3 LCPUFAs induced autophagy via modulating the phosphatidylinositol 3-kinase (PI3K)/AKT/mTOR pathway.^51^^,^^74^ Nayeong Kim et al. found that pretreatment with rapamycin increased DHA-induced cleaved-poly(ADP-ribose) polymerase and LC3-II levels in A549 cells, suggesting that autophagy and apoptosis induced by DHA were associated with the inhibition of mTOR. Further researches found that ω-3 LCPUFAs triggered apoptosis and autophagy through the AMP-activated protein kinase (AMPK) and PI3K/AKT signaling pathways.^48^ Qinghua Yao et al. demonstrated that DHA and EPA disrupted the formation of autophagosomes via activating the AKT/mTOR pathway, thereby prematurely lowering autophagic signaling and downregulating Beclin-1 levels, thus inducing lung cancer cell death.^51^ Yuanqin Yin et al. also discovered that the inhibition of AKT phosphorylation at the serine-473 site by DHA induce lung cancer cell death, further supporting the important roles of AMPK and AKT pathways.^49^^,^^51^ In general, the above findings demonstrated that ω-3 LCPUFAs contributed to the death of lung carcinoma cells, indicating that ω-3 LCPUFAs could potentially be a beneficial agent for treating NSCLC in humans and that it is important to explore therapeutic strategies using ω-3 LCPUFAs as nutritional supplements.

### ω-3 long-chain polyunsaturated fatty acids suppress lung cancer via inhibiting cell metastasis

Approximately 45% NSCLC recur after complete resection, mainly caused by metastasis.^49^ Hence, preventing and treating metastasis of lung cancer has great significance.^49^ Currently, the main therapeutic options for tumor metastasis are inhibiting neovascularization, blocking epithelial mesenchymal transformation, and applying effective inhibitors.^75^ Previous researches showed that ω-3 LCPUFAs inhibited tumor metastasis via inhibiting neovascularization and blocking epithelial mesenchymal transformation.^75^ Mehboob Ali et al. discovered that DHA could increase the level of actin-binding proteins (ABP) to limit the migration of A549 cells.^76^ In addition to ABP, matrix metalloprotein (MMP) enzymes are also critical proteins for tumor invasion and metastasis, promoting cancer cell metastasis via degrading the extracellular matrix.^77^ DHA was reported to inhibit the COX-2/PGE2/NF-κB/MMP signaling pathway to suppress cancer cell metastasis.^78^ More importantly, recent studies found that DHA regulated the expression of MMP-9 and cancer cell invasion via modulating MAPK and PPAR-γ/NF-κB pathways.^77^ Yuanqin Yin et al. also reported that DHA suppressed A549 cell migration and invasion via downregulating MMP-9, human enhancer of filamentation 1 and VEGF.^49^

The ratio of ω-3 and ω-6 LCPUFA consumption has a significant effect on the risk and progression of tumors. A recent study discovered that ω-6 LCPUFA enriched diet regulated NF-B-p65 levels and activated the transcription factor Yin Yang 1 to upregulate COX-2 and transforming growth factor (TGF)-β, ultimately encouraging a more aggressive tumor phenotype, while ω-3 LCPUFA could reverse these effects.^79^ The specific mechanisms that participate in the metastasis of carcinoma are still unclear, but the above researches indicated that ω-3 LCPUFA as nutritional therapy might be an effective therapeutic strategy.

### ω-3 long-chain polyunsaturated fatty acids modulate lipid raft structure and membrane functions

ω-3 LCPUFAs were found to be associated with phospholipid membranes, causing changes in membrane fluidity and lipid raft structure. Such membrane changes affect the membrane receptor activity,^80^ downregulating oncogenes and suppressing signaling pathways that are engaged in cancer cell survival.^23^^,^^81^^,^^82^ The involvement of lipid rafts on phospholipid membranes modulates T cell activation and monocyte functions.^42^^,^^80^ Kristina Rogers et al. showed that DHA can alter epidermal growth factor receptor (EGFR)-associated signaling by disrupting its lipid raft binding, thus enhancing the efficacy of EGFR inhibitors and inhibiting tumor growth in lung cancer.^83^Given the fact that fatty acid can regulate the structure and function of lipid rafts, it is possible that ω-3 LCPUFAs decelerate tumor progression via changing the structure of their membranes.^42^^,^^80^^,^^84^ In addition, several anti-tumor agents are designed to alter the lipid raft’s protein content to achieve their effects.^85^

Previous studies demonstrated that the regulation of EPA content in cell membrane phospholipids could modulate the body’s response to the inflammatory state because EPA and DHA affect inflammation processes that correlate with alterations of fatty acid composition in cell membrane.^81^ These findings hold great significance for researches on therapeutical strategy design of ω-3 LCPUFAs as dietary substances. At present, the mechanisms of lipid raft alteration by ω-3 LCPUFAs in lung cancer are not fully understood, and the underlying mechanisms are still to be investigated.

### ω-3 long-chain polyunsaturated fatty acids suppress lung cancer through their anti-inflammatory effects

Tumor inflammatory microenvironment is important for tumor survival,^23^ and inflammation acts as a cancer trigger to facilitate cancer invasion.^86^ While ω-6 LCPUFAs are the precursors of pro-inflammatory mediators, ω-3 LCPUFAs are widely recognized to have anti-inflammatory properties. Previous studies revealed that taking ω-3 LCPUFAs supplements at a dose of 3.6 g/day reduced the inflammatory markers C-reactive protein (CRP) and PGE2.^87^ Therefore, the benefits of ω-3 LCPUFAs to prevent and treat cancers attract increasing attentions.^43^

#### ω-3 long-chain polyunsaturated fatty acids and inflammatory factors

Tumor progression is regulated by various pro-inflammatory, such as interleukin (IL)-6 or TNF-α, and anti-inflammatory factors, such as IL-10 and TGF-β. In the clinic, the levels of CRP in the blood are usually used as an indicator for the detection of systemic inflammation.^4^^,^^88^ In a nicotine-derived nitrosamine ketone-induced lung cancer model, Ingrid Elisia et al. discovered that incorporating ω-3 LCPUFAs into a low carbohydrate diet (15% straight-chain starch/soy/fish oil, approximately the same ω-6 LCPUFAs content but with 47.3 g/kg ω-3 LCPUFAs and an ω-6/ω-3 ratio is 0.7) significantly reduced IL-6 levels in the plasma, resulting in less lung nodules and slowing tumor growth.^10^

#### ω-3 long-chain polyunsaturated fatty acids and cyclooxygenase

Besides inflammatory factors, COX is considered as a powerful pro-inflammatory and pro-cancer agent as well.^23^^,^^89^ ω-3 LCPUFAs were reported to lower the COX-2 expression and the synthesis of PGE2, showing the potential for anti-inflammatory or anti-proliferative activity.^58^^,^^90^ ω-3 LCPUFAs compete with ω-6 LCPUFAs as enzymatic substrates for COX and lipoxygenase, and their metabolites act as precursors of resolvins and protectins to terminate the inflammatory process, resulting in providing protection against cancer initiation.^91^ E-series resolvins were recently reported to suppress inflammation and angiogenesis of tumor microenvironment in lung cancer, slowing tumor growth and improving clinical responses to chemotherapy.^92^ Mayra Montecillo-Aguado et al. detected lower expression levels of COX-2 in tumors of mice on an ω-3 vs. ω-6 LCPUFA rich diet, demonstrating that high dietary intake of ω-6 LCPUFA increased malignancy in neoplastic cells,^79^ and that ω-3 LCPUFAs exert anti-inflammatory and anti-tumor effects via reducing COX activity.^82^ Given that COX-2 is overexpressed in approximately 70% lung adenocarcinomas,^58^^,^^93^^,^^94^ diets such as LCPUFA supplements may provide a naturally effective treatment or prevention for lung cancer, therefore we encourage increased ω-3 LCPUFAs intake and reduced ω-6 LCPUFAs consumption to improve the ω-3/ω-6 ratio, which may be beneficial for the prevention and treatment of lung cancer.

#### ω-3 long-chain polyunsaturated fatty acids and nuclear factor-κB

NF-κB is also closely associated with inflammation, and its continued activation leads to deregulated cell growth and promoted carcinogenesis.^95^ In cell cultures and animal models, NF-κB is activated and upregulated during inflammation, thus boosting the expression of several pro-inflammatory cytokines such as IL-1β, IL-6, COX-2, and TNF-α and altering tumor inflammatory microenvironment to promote tumor cell survival.^96^^,^^97^ Payungsak Tantipaiboonwong et al. found that PSO and rosemary acid decreased NF-κB expression induced by TNF-α in lung cancer cells and mouse models, leading to reduced expression of IL-1β, IL-6, IL-8, TNF-α, and COX-2.^57^ Caroline Morin et al. also found that treatment with the synthetic DHA derivative CRBM-0244 caused a reduction in TNF-a-triggered NF-kB activation and overexpression of COX-2 in the lung.^98^ In addition, EPA and DHA also impacted NF-κB activation through another transcription factor with anti-inflammatory effects, PPARγ.^81^ DHA is also considered as a natural ligand of PPARγ,^99^ and the EPA derivatives such as PGD3 and 15-deoxy-d12,14-PGJ3 induce the anti-inflammatory adipokine lipocalin production via activating PPARγ.^81^^,^^100^ Upon activation by ω-3 LCPUFAs, PPARγ directly affects the NF-κB pathway and downregulates the level of pro-inflammatory factors such as MCP-1, IL-6, and TNF-α, so as to suppress neoplasia development.^37^^,^^101^ Currently, PPARγ agonists were investigated in Phase I/II clinical trials for their anti-tumor effects.^37^ Moreover, LCFUFA lipid mediators (e.g., resolvins) were found to inhibit NF-κB and exhibit important anti-inflammatory and cytoprotective effects.^13^^,^^102^ Although the NF-κB pathway regulates cytokines that alter the immune microenvironment to influence tumor growth, however it also affects the normal immune responses. Therefore, the exact mechanisms are required to be examined in more depth to balance the relationship between the both.

In summary, ω-3 LCPUFAs prevent and inhibit cancers via producing anti-inflammatory effects through various pathways. However, the influence of inflammation on most cancers is double-edged. Meanwhile, cancer cells affect the process of inflammation in a backward. The essence of inflammation-targeted cancer therapy is to promote cancer-inhibiting inflammation and suppress cancer-promoting inflammation. The main challenge is maintaining the balance of good and bad inflammatory effects,^103^ so a better understanding on the interactions between ω-3 LCPUFAs and inflammation in lung cancer is critical.

### ω-3 long-chain polyunsaturated fatty acids increased tumor therapeutic efficacy

Besides radiotherapy and surgery, drug-based chemotherapy, targeted therapy, and immunotherapy are indispensable therapeutic approaches for lung cancer treatment and the primary recommendation for treatment.^3^ However, therapeutic resistance toward these drugs is a critical problem contributing to poor outcomes. Platinum-based drugs are the basic drugs for lung cancer chemotherapy, but resistance is also unavoidable.^104^ Now many studies have indicated that increasing ω-3 LCPUFAs intake significantly benefits chemotherapy outcomes,^33^^,^^37^ Caroline Morin et al. discovered that DHA could synergize with carboplatin to inhibit tumor growth via suppressing EGFR and ERK signaling pathways in the xenograft mouse model.^60^ Another research also demonstrated that selenium yeast combined with fish oil was capable to increase the sensitivity of A549 cells toward cisplatin.^105^ Apart from platinum-based chemotherapeutic agents, compared with the drugs alone, Shougang Jiang et al. also demonstrated a better tumor metastasis inhibitory effect of DHA and docetaxel in mice, which effectively avoided the metastasis of lung cancer to bone.^62^

Except for cytotoxic chemotherapy, targeting the EGFR is a milestone in targeted therapy for lung cancer, and the use of targeted agents has dramatically improved the survival and quality of life of patients with lung cancer; however, limited by acquired resistance, this targeted therapy is also unable to maintain long-term efficacy.^106^^,^^107^ Chien-Huang Liao et al. combined ω-3 LCPUFAs with selenium and discovered it could increase sensitivity to gefitinib in lung cancer by modulating the endoplasmic reticulum stress response.^32^ Xuansheng Ding et al. also showed that DHA can act as a sensitizer of gefitinib and strengthen its efficacy.^61^

Immunotherapy has fundamentally changed the treatment paradigm of lung cancer and has become one of the standard treatments for lung cancer; but equally, immunoresistance is an urgent issue that needs to be addressed.^108^ Han Zhang et al. first discovered that DHA could reduce the expression of programmed cell death ligand 1 (PD-L1) *in vivo* and *in vitro* by inducing ubiquitin-proteasome degradation, resulting in enhancing the immune system and inhibiting tumor growth.^64^ Subsequently, another recent study has also demonstrated that PUFA enrichment on membrane phospholipids increases membrane fluidity, thus decreasing the binding of PD-L1 and programmed cell death protein 1 (PD-1), and increasing the effect of ICIs.^63^

As indispensable FAs in the human body, LCPUFAs have emerged as a dietary supplement for cancer prevention due to their significant anti-inflammatory effects, multiple anticancer mechanisms, and non-toxicity; at the same time, LCPUFAs have also emerged as a novel combination for lung cancer treatment due to their abundant anticancer effects. Importantly, besides proving to be effective in preclinical models, LCPUFAs have also been demonstrated to improve the quality of life and outcomes in the clinic, and we will discuss this in the next section.

## Clinical effects of ω-3 long-chain polyunsaturated fatty acids on the treatment of patients with lung cancer

With nutrition being a vital aspect in multimodal cancer management,^109^ using ω-3 LCPUFAs for nutritional therapy to prevent different types of cancer, including lung cancer, has been a hot topic in recent years. Currently, ω-3 LCPUFAs are regarded as medicinal nutrients that regulate signaling pathways, decrease inflammatory responses, and enhance chemotherapeutic efficacy, thus improving the overall survival of patients with lung cancer.^6^ According to estimates, approximately 10–20% of patients with lung cancer perished from malnutrition rather than the tumor itself,^109^ and 85% of these patients suffer from the anorexia-cachexia.^110^ The distinctive characteristic of patients with cachexia is loss of skeletal muscle and adipose tissue specifically, resulting in the malnutrition of patients. There is no effective medical or approved pharmacological treatment that can fully reverse cachexia.^111^ Here we summarize the clinical trial results of ω-3 LCPUFAs interventions over the last decade in order to provide clinical evidence (Table 2) for the ω-3 LCPUFAs supplementation into clinical practice and the nutritional care protocols.

**Table 2:** The effects of ω-3 LCPUFAs in clinical use for patients with NSCLC

Cancer (Stage)	N (Int/Cnt)	Basic treatment	Int and Cnt	Treatment duration	Major outcome	Reference
NSCLC (III or IV)	46 (31/15)	Chemotherapy	Int = EPA+DHA 2.5 g/d Cnt = N/A	6 weeks	Chemotherapy response rate and clinical benefit ↑	Murphy et al.^112^
NSCLC (III or IV)	40 (16/24)	Chemotherapy	Int = EPA 2.2 g/d Cnt = SoC	10 weeks	Body weight and muscle mass ↑	Murphy et al.^113^
NSCLC (Advanced)	27 (13/14)	Chemotherapy	Int = EPA 510 mg/d+DHA 340 mg/d CNT = placebo	66 days	Body weight ↑ HNE, ROS, CRP and IL-6 ↓	Finocchiaro et al.^114^
NSCLC (III)	40 (20/20)	Chemotherapy+ radiotherapy	Int = EPA2.02 g/d+DHA 0.92 g/d Cnt = isocaloric	6 weeks	Life quality, physical activity level, handgrip strength ↑	van der Meij et al.^115^
NSCLC (27.5%) and other cancers	69 (35/34)	Chemotherapy+ surgery (58%, before chemo)	Int = EPA 3.3 g/d+EPA 2.2 g/d Cnt = 600 kcal	4 weeks	CRP ↑ (short intervention, abandon treatment)	Pastore, et al.^116^
NSCLC (III b or IV)	92 (46/46)	Chemotherapy	Int = ONS-EPA Cnt = isocaloric	8 weeks	body weight, energy and protein intake ↑ CRP, TNF-α, NLR, PLR ↓	Sánchez-Lara et al.^117^
NSCLC	58 (31/27)	Surgery	Int = immune modulator	preoperative nutrition for 10 days	albumin loss, complications ↓	Kaya, et al.^118^
NSCLC (Advanced)	137 (77/60)	Chemotherapy	Int = EPA510 mg/d+DHA200 mg/d Cnt = N/A	6 weeks	CRP, IL-6 ↓	Lu et al.^4^
NSCLC	58 (29/29)	Standard treatments	Int = EPA 1.6 g/d+ DHA 0.8 g/d	12 weeks	body weight ↑ CRP, TNF-α ↓	Cheng et al.^119^

### ω-3 long-chain polyunsaturated fatty acids in clinical applications

Cancer nutritional therapy focuses on modulating tumor responses, as well as attenuating the toxicity and pathological alterations in the host organism.^120^ Previous studies suggested that both EPA and DHA could strengthen the effectiveness of cytotoxic agents against tumors without additional toxicity to the host.^120^^,^^121^ Epidemiological researches indicated that the consumption of LCPUFAs was significantly and negatively related to lung cancer risk and mortality.^58^^,^^122^

Due to their potential nutritional support and anti-inflammatory and anti-tumor roles, ω-3 LCPUFAs are potentially useful therapeutic agents either by themselves or in combination with other therapeutical strategies.^53^ A meta-analysis in 2022 demonstrated that ω-3 LCPUFAs benefited nutritional status and modulated inflammatory markers in patients with lung cancer treated with chemoradiotherapy.^123^ Clinical practice revealed that ω-3 LCPUFAs are advantageous for patients with NSCLC with chemotherapy, particularly platinum-based ones.^6^ In a clinical trial, Rachel. Murphy et al. found that the addition of EPA plus DHA at 2.5 g/day dramatically enhanced response rates to the chemotherapy without toxicity profiles, suggesting that ω-3 LCPUFAs benefited advanced NSCLC with platinum-based chemotherapy.^112^ BS van der Meij et al. reported that oral nutritional supplements of ω-3 LCPUFAs positively affected pulmonary functions and quality of life in patients with NSCLC receiving multimodal therapy.^115^ Moreover, Sanchez-Lara et al. revealed that EPA-containing nutritional supplements alleviated appetite and neuropathy in patients with NSCLC with chemotherapy.^6^^,^^117^

In addition to benefiting chemotherapy, Seyda Ors Kaya et al. showed that pre-operative nutritional support of ω-3 LCPUFAs was associated with less post-operative albumin loss, fewer post-operative complications, and shorter hospital time for patients with lung cancer.^118^ Ultimately, the nutritional status, life quality, and treatment tolerance of patients can all be maintained or improved with nutritional therapy.^115^^,^^124^ Carlos Déniz et al. also discovered the ratio of preoperative ω-6/3 LCPUFAs was related to postoperative complications, and adding ω-3 LCPUFAs through diet may be able to prevent postoperative air leak.^125^ Although the precise mechanisms causing these benefits have not yet been identified, these researches demonstrated the beneficial effects of ω-3 LCPUFAs supplement. When combined with currently used anti-tumor agents, ω-3 LCPUFA improves the benefits of these therapeutical strategies and decreases their nutritional adverse effects, reflecting that ω-3 LCPUFA has the potential to be an effective adjuvant for lung cancer treatments.

### Roles of ω-3 long-chain polyunsaturated fatty acids in cachexia

Currently, although surgery is still the first and standard choice for patients with NSCLC,^126^ less than 30% of patients receive this treatment.^127^ Radio-chemotherapy is widely applied for those not suitable for surgery. However, patients often have esophagitis, appetite loss or other medical conditions after radio-chemotherapy,^6^ and these can impair the patient’s nutrition and lower their life quality. Approximately 45–69% of patients with lung cancer treated with chemotherapy or other treatments are malnourished, resulting in a poor response to therapies.^6^^,^^7^ If this condition is not well treated, it will lead to cancer cachexia.^4^ Despite the improvement of various therapeutical options for lung cancer, cachexia is the leading contributor to death.^128^ Therefore, it is important and meaningful to avoid the loss of nutrition in patients with lung cancer.

Currently, ω-3 LCPUFAs are recognized as pharmacological nutrients that reduce inflammatory responses, improve chemotherapy efficacy, and improve the nutritional status of patients, thus improving the overall survival of patients with cancer.^24^ According to statistics, 20–80% of patients require dietary supplementation to maintain their nutritional status after diagnosis.^24^^,^^129^ In 2017, the ESPEN suggestions for nutritional and metabolic management recommended ω-3 LCPUFAs as nutritional components for the treatment of cachexia in patients with cancer.^6^^,^^8^ In a research exploring its feasibility and efficacy in patients with NSCLC, dietary intake of fish oil (2 g EPA or DHA per day) significantly induced skeletal muscle gain (*p* < 0.02), indicating that a multimodal intervention is feasible in phase I antitumor therapy for lung cancer.^9^

The inflammatory factors are associated with the regulation of muscle proteins, accelerating the cachexia process, and cytokines are crucial in the development of cancer cachexia.^130^ Since ω-3 LCPUFAs have good anti-inflammatory properties, their regulation of pro-inflammatory cytokines becomes important for their anti-cachectic effects.^130^ Patients with NSCLC treated with ω-3 LCPUFAs for 6 weeks showed better CRP and IL-6 levels.^4^ In another study, 92 advanced patients with NSCLC were randomly administered oral nutritional supplements with EPA (ONS-EPA) or equal calorie dietary only. It was found that the energy and protein intakes of the ONS-EPA group were considerably greater than the control (*p* < 0.001). The patients receiving ONS-EPA increased lean body mass by 1.65 kg, while those in the control group reduced by 2.06 kg (*p* < 0.01). Further studies revealed that ONS-EPA significantly downregulates CRP and TNF-α, neutrophil/lymphocyte ratio and platelet/lymphocyte radio, as well as pro-inflammatory status, indicating that intake of EPA significantly benefited the patients with NSCLC to absorb energy and proteins and to improve the body composition, which prevented cachexia development.^117^

Overall, ω-3 LCPUFAs and their metabolites may modulate key pathways of cancer complications, suggesting their promising effects on tumor treatments as natural dietary agents. Further investigations should be designed to examine the underlying mechanism of how they reduce cancer development and the incidence of cancer cachexia.

## Summary

Lung cancer is one of the most frequently diagnosed cancers, and its high mortality rates require continued researches. ω-3 LCPUFAs as nutritional supplements and prescription medication are largely accepted all over the world. In the past decades, more and more experiments are supporting the role of LCPUFAs in the progression of lung cancer, in addition to anti-inflammatory effects, LCPUFAs also inhibit the development of tumors by altering the lipid rafts of cell membranes, regulating tumor autophagy and apoptosis, and inhibiting cell proliferation and migration, and so forth. Crucially, both ω-3 LCPUFAs and their derivatives seem to exhibit cytotoxic effects mainly against carcinoma cells, but not normal cells, while it is possible to enhance tumor cell sensitivity to drugs when combined with other pharmaceutical agents, such as ICIs, targeted inhibitors, and other chemotherapeutic agents. Certainly, as mentioned earlier, LCPUFAs *in vivo* are affected by a variety of factors, besides anticancer effects, LCPUFAs also promote tumorigenesis when redox imbalance occurs,^122^ as well as the richness of the species and lipid products of LCPUFAs, our current knowledge is still insufficient, so in the future, we need to investigate more about the specific mechanisms of LCPUFAs in lung cancer and provide guidance for the clinical research.

Notably, although the current preclinical evidence basically supports the tumor inhibitory effect of LCPUFAs, especially ω-3 LCPUFAs, the controversy regarding the relation between LCPUFAs and lung cancer risk still remains, and the discrepancy between the results could be the source of ω-3 LCPUFAs, the type of ω-3 LCPUFAs (EPA or DHA), the ratio of ω-3 to ω-6 LCPUFAs, and the lifestyles in different regions, and so forth, So we still need additional clinical research to demonstrate the relationship between ω-3 LCPUFAs and lung cancer in the future. According to the current clinical results and meta-analysis, we consider that for patients with lung cancer, the supplementation of ω-3 LCPUFAs should be at a dose of 2–5 g/day,^19^^,^^21^^,^^131^ with EPA content higher than that of DHA^117^; and depending on the average duration of the current clinical studies, we suggest that it is preferable to continue the supplementation of LCPUFAs for more than 2 months; in this way, patients with cancer could obtain certain clinical benefits such as suppression of the inflammatory response, better nutritional status, improved quality of life, and potentially altered treatment effects. For pre-cancerous prophylaxis, we recommend consuming foods rich in ω-3 LCPUFAs (preferably EPA over DHA), such as cod, halibut, herring oil, and lobster.^132^ Due to the limited amount of research available, we cannot extrapolate the superiority of EPA over DHA, and the specific clinical dosages and durations, without definitive clinical evidence.

According to the ICTRP (https://trialsearch.who.int) database, several studies on LCPUFAs and lung cancer are currently underway (NCT06531460, NCT04965129), and we expect more evidence in the future to prove LCPUFAs and lung cancer treatment, providing a more accurate direction for individualized nutrition.

## Acknowledgments

This study was supported by 10.13039/501100001809National Natural Science Foundation of China (82473253 and 82471098), Central Government-Guided Local Science and Technology Development Project (2024ZYYD001), Translational Medicine and Interdisciplinary Research Joint Fund of 10.13039/501100016359Zhongnan Hospital of Wuhan University (ZNJC202007), and Medical Science and Technology Innovation Platform Construction, Support Project of 10.13039/501100016359Zhongnan Hospital of Wuhan University (PTXM2022009).

## Author contributions

Conceptualization, J.L., S.P., Z.J., C.X., and Y.G.; validation: J.L., S.P., Q.W., and M.Z.; resources: Y.Z., Y.Yuan., M.X., Z.H., and Y.Yan; writing-original draft preparation, J.L., P.S, and Z.J.; writing-review and editing: all authors; visualization, J.L., S.P., Z.J., Y.T., and J.T.; supervision, C.X. and Y.G.; project administration, C.X. and Y.G. All authors have read and agreed to the published version of the article.

## Declaration of interests

The authors declare no conflict no competing interests.
